# *Salmonella* empyema: a case report

**DOI:** 10.1007/BF03371465

**Published:** 2015-12-01

**Authors:** Shivanshan Pathmanathan, Suminda Welagedara, Petra Dorrington, Siddharth Sharma

**Affiliations:** 115 Department of Medicine, Gold Coast University Hospital, 1 Hospital Boulevard, Southport, Queensland 4215 Australia; 215 Department of Microbiology, Gold Coast University Hospital, Southport, Queensland Australia; 315 School of Medicine, Griffith University, Southport, Queensland Australia

**Keywords:** Salmonella, enterica, empyema, pleural, pulmonary

## Abstract

Non-typhi *Salmonella enterica* infection rarely presents as a pleural empyema, with only 31 cases published in the literature over the last century. We report a case of an 85-year-old female with worsening shortness of breath and pleuritic chest pain, and a chest radiograph showing a right-sided pleural effusion. Thoracocentesis revealed *Salmonella enterica* serovar Typhimurium to be the causative organism. This was on a background of recurrent pleural effusion secondary to congestive heart failure, with thoracocentesis one month previously showing a transudative picture. This case highlights the possibility of *S. enterica* as a differential diagnosis in the management of pleural effusions.

## 1. Introduction

*Salmonella* spp., first described in the 1880s by Salmon and Smith [1,2], are Gram-negative, non-spore-forming, facultative anaerobic bacilli of the family *Enterobacteriaeceae*. *Salmonella enterica* serovars Typhi and Paratyphi produce a spectrum of clinical presentations including enteric fever. Non-typhi *S. enterica* serovars most commonly present with gastroenteritis [3]. Extra-intestinal manifestations include bacteraemia and focal infections ranging from endovascular arteritis, endocarditis, septic arthritis, osteomyelitis, urinary tract infection to splenic abscess. Non-typhi *S. enterica* serovars rarely cause pleuropulmonary disease, in particular pleural empyema. Only 1 case of non-typhi *S. enterica* empyema has been reported in Australia [4]. This case report presents a case of unilateral empyema caused by *Salmonella enterica* serovarTyphimurium, and a review of the literature.

## 2. Case report

An 85-year-old female of English heritage presented with a 3-day history of increasing right-sided pleuritic chest pain associated with increased shortness of breath and increased non-productive cough. There was no fever or increased sputum production. The patient did not report diarrhoea, abdominal or flank pain on admission, or in the 12 months leading to admission. Significant past medical history included left ventricular heart failure complicated by previous presentations with right-sided pleural effusions requiring thoracocentesis. Thoracocentesis 1 month before this admission had shown a transudative picture with no bacterial growth on culture. Other clinical history included ischaemic heart disease, severe mitral valve stenosis, atrial fibrillation, hypertension, mild neutropenia (1.25 × 10^9^/l), pending bone marrow aspirate, and *Streptococcus gallolyticus* subsp. *gallolyticus* infective endocarditis in 2010. There was no suggestion of prior *S. enterica* infection, cholelithiasis or urolithiasis. There was no history of travel outside Australia in the preceding 12 months.

Physical examination revealed an oxygen requirement (4 litres to maintain saturations greater than 95%), respiratory rate of 16 breaths/min, tachycardia (100–120 beats/min, irregular), normal blood pressure (120/70 mmHg) and afebrile. Cardiovascular examination revealed a jugular venous pressure elevated at 7 cm and a Grade 3 pansystolic murmur over the mitral region, radiating to the axilla. Respiratory examination revealed dullness to percussion in the right middle to lower zones, with reduced breath sounds noted in the same area. The abdomen was soft on palpation and no tenderness was elicited. There was bilateral pitting oedema to the level of the knees. Initial pathology revealed a normal white blood cell count of 2.7 × 10^9^/l with 46% (1.25 × 10^9^/l) neutrophils and 30% (0.82 × 10^9^)/l lymphocytes. C-reactive protein (CRP) was elevated at 268 mg/l.

**Figure 1:**
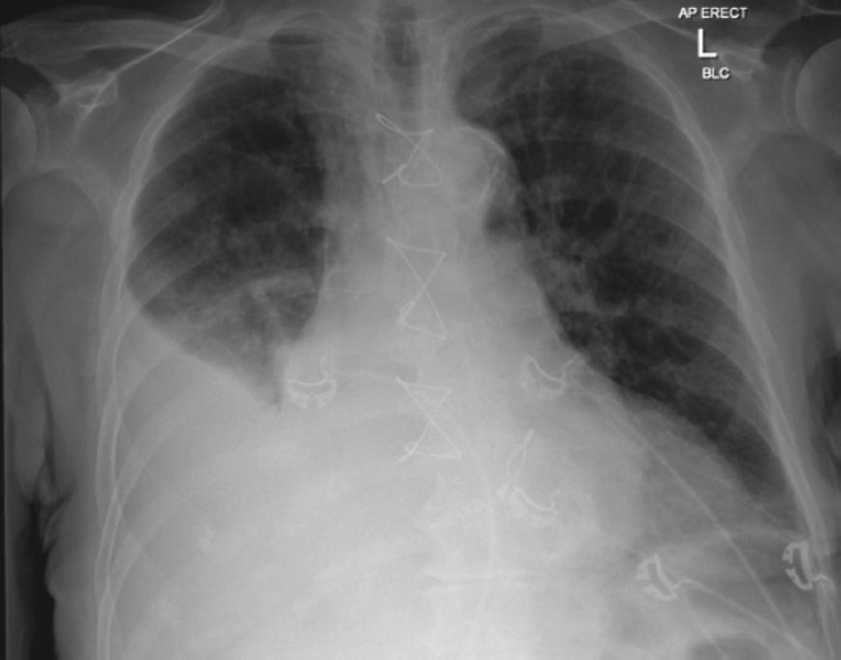
Chest radiograph on presentation showing evidence of right-sided pleural effusion

The chest radiograph revealed opacification of the lower two-thirds of the right hemithorax, with loss of the right costophrenic angle. Underlying collapse was also noted. The chest radiograph can be viewed in Figure 1. A thoracocentesis was subsequently performed. Analysis of pleural fluid revealed a white cell count of 1,350 × 10^6^/l, a protein concentration of 35 g/l, and a lactate dehydrogenase level of 590 U/l. These values were consistent with exudate. The pH of the sample was 7.1, indicating empyema. Gram-negative bacilli were seen in a Gram-stained film of the fluid. A second thoracocentesis was performed 2 days later, again showing Gram-negative bacilli. Subsequent cultures grew *S. enterica* serovar Typhimurium. Three sets of blood cultures and 2 stool cultures taken prior to starting antibacterial therapy were negative for *S. enterica*.

The intended management of the patient included insertion of a chest tube (tube thoracostomy), and a 4-to 6-week course of ceftriaxone, depending on clinical response. However the patient declined a chest drain, and wished to cease ceftriaxone after two days. The patient received palliative care aiming for symptom control. Challenges included the management of severe right sided pleuritic chest pain, dyspnoea and nausea, requiring continuous subcutaneous infusion of fentanyl, haloperidol and maxalon. Hydromorphone was used for breakthrough analgesia. The patient passed away comfortably in the palliative care unit 5 days after stopping the antibiotics.

### 2.1 Ethics statement

This case report complies with the current policies of Gold Coast Hospital and Health Service, Gold Coast, Queensland, Australia (GCHHS) for a deidentified patient who is deceased. Patient consent for publication was obtained and recorded in the electronic medical records. For the purpose of publication the HREC reference number is: HREC/15/QGC/190.

## 3. Discussion

A literature review published by Crum [5] only identified 28 cases of *S. enterica* empyema up to 2005. The literature review included adult and paediatric patients and excluded empyema caused by *S. enterica* serovars Typhi and Paratyphi. From 2005 to 2015, 2 further cases of non-typhi *S. enterica* empyema have been identified in the literature [6] [7]. Only 1 published case has been identified in Australia (1991) [4]. The patient was a 70-year-old male with a background of lymphoma in remission who presented with a 6-week history of chest pain, and was found to have *S. enterica* serovar Typhimurium. The patient was managed with intravenous ampicillin, and decortication. The underlying cause was not determined. *S. enterica* serovar Typhi also rarely presents with pleural empyema, with only 7 cases being reported in the literature, predominantly in the paediatric population [8] [9].

Although a rare diagnosis, trends in co-morbidities and symptoms have been reported. It is common for patients to be immunosuppressed secondary to co-morbidities such as human immunodeficiency virus infection, diabetes, malignancy, iatrogenic (prednisone, azathioprine, chemotherapy), iron overload, and chronic renal insufficiency [4] [5] [7] [10] [11] [12]. Pre-existing lung disease has also been noted to be present in 38% of the patients, suggesting a propensity to adhere to damaged lung tissue [5] [13] [14] [15]. The patient reported in this study had a combination of prior lung disease with prior pleural effusion, and immunosuppression secondary to a Grade 1 neutropenia. Although the patient did not have a significant travel history, other risk factors would include travel to endemic areas such as India, South-East Asia and Africa [16].

There was no history of recent or prior *Salmonella* infection in this patient, although prior reports have shown only 33% of patients had a history of gastrointestinal illness [5]. A second interesting feature is that she had presented 4 weeks prior with similar symptoms. Thoracocentesis was performed and showed a transudative picture, attributed to congestive heart failure. Therefore, pleural fluid chemistry can change, and results of recent pleural fluids sampling should be used with caution in the context of changed clinical scenario. This is particularly true for patients with risk factors such as heart failure or lung cancer who may present repeatedly with pleural effusions. Non-typhi *S. enterica* serovars empyema commonly presents with a leukocytosis [5]. The lack of leukocytosis in this case can be attributed to the immunosuppression secondary to neutropenia and possible bone marrow involvement. In this case, CRP was elevated and would have been the more appropriate method to monitor the inflammatory response.

Transmission of non-typhi *Salmonella* predominantly occurs through infected food products such as eggs and dairy products. Most commonly, patients present with local symptoms of gastroenteritis [3]. The hypothesised pathogenesis of focal infections such as empyema includes bacteraemia with subsequent seeding in patients presenting with positive blood cultures for *S. enterica* [10] [12] [17]. Other causes include seeding from nearby infection such as the spleen and pancreas, with subsequent transdiaphragmatic tracking [7] [18] [19] [20] [21] [22] [23]. Therefore, isolation of an enteric organism, such as *Salmonella*, from a pleural effusion should prompt clinicians to exclude an intra-abdominal source of infection.

Our observations in this case report are consistent with previous cases, which showed no preceding history of *Salmonella*, blood cultures were negative and stool cultures were negative [5]. In these cases, only 30% had positive blood cultures, while 39% had positive stool cultures. It is suspected that non-typhi *Salmonella* may be dormant in the reticulo-endothelial system, with subsequent re-activation and haematogenous spread [24]. It should also be noted that blood cultures are regularly negative in *Salmonella* bacteraemia due to low bacterial load [25]. The sensitivity of detection by blood culture also declines with longer duration of the illness [26].

Prior case reports used an extensive range of antibiotics in the treatment of *Salmonella* empyema [27]. Traditionally, ampicillin, chloramphenicol, and cotrimoxazole were used in the management of non-typhi *Salmonella* infections. However, in light of increasing resistance, third-generation cephalosporins are commonly empirically used until further susceptibility to quinolones is available. The effective utilisation of antibiotics improves outcomes [24].

In conclusion, *Salmonella* empyema is an uncommon presentation, with only 31 case reports published in the last century. It can be a difficult diagnosis, with a lack of gastrointestinal symptoms, a lack of raised leucocytes, or a lack of positive stool or blood cultures to assist with diagnosis. Therefore, although rare, *Salmonella* as a cause of pleural effusion should remain a differential diagnosis.
